# Supplementary figures and images for: Genome-Wide Identification of Ginkgo biloba SPL Gene Family and Expression Analysis in Flavonoid Biosynthesis and Water Stress
Source: Int J Mol Sci. 2025 May 21;26(10):4932. doi: 10.3390/ijms26104932 (PMC12112263; doi:10.3390/ijms26104932)

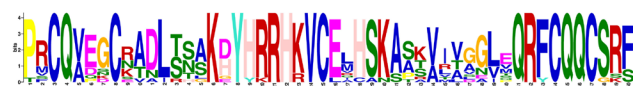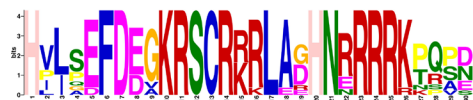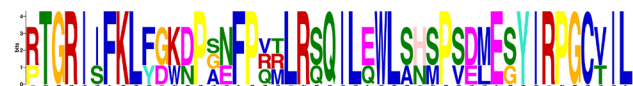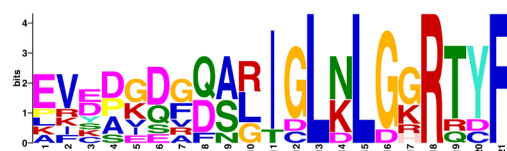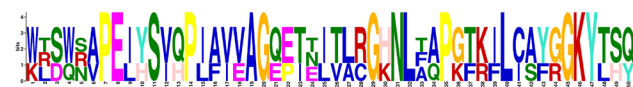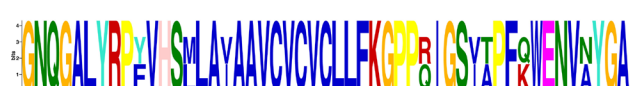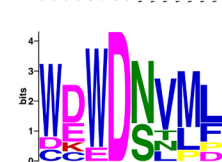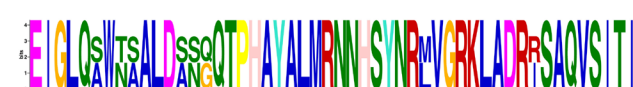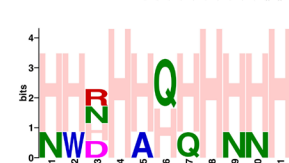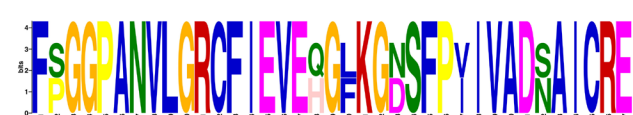

| E-value ? | Sites ? | Width ? |
|-----------|---------|---------|
| 4.3e-383  | 13      | 50      |
| 1.4e-151  | 10      | 30      |
| 9.2e-034  | 3       | 47      |
| 6.3e-030  | 8       | 21      |
| 6.7e-021  | 3       | 50      |
| 4.7e-016  | 2       | 49      |
| 7.2e-014  | 8       | 8       |
| 1.1e-013  | 2       | 50      |
| 8.5e-010  | 4       | 11      |
| 5.0e-009  | 2       | 37      |

Supplement: Supplementary file 1 [file ijms-26-04932-s001.zip › ijms-3619952-supplementary/Figure S1.pdf]
